# Complement in Human Disease

**DOI:** 10.1155/2013/920474

**Published:** 2013-01-17

**Authors:** Michael A. Flierl, Daniel Rittirsch, Markus S. Huber-Lang, Philip F. Stahel

**Affiliations:** ^1^Department of Orthopaedic Surgery, School of Medicine, University of Colorado, Aurora, CO 80045, USA; ^2^Division of Trauma Surgery, Department of Surgery, University Hospital Zurich, 8006 Zurich, Switzerland; ^3^Department of Trauma-, Hand-, and Reconstructive Surgery, University Hospital Ulm, 89081 Ulm, Germany; ^4^Department of Orthopaedic Surgery, Denver Health Medical Center, School of Medicine, University of Colorado, Denver, CO 80204, USA

As part of innate immunity, the complement system embodies the “first line of defense” to various initial insults, including trauma, infection, hemorrhage, ischemia, and autoimmunity. While of beneficial intention, excessive complement activation can inadvertently damage healthy host tissues and thereby exacerbate the initial pathological events by causing an “innocent bystander effect.” 

“Complementology” represents a rapidly evolving field. Recent scientific efforts have identified novel complement activation pathways and select complement-inhibiting compounds, which have been shown to ameliorate myriad autoimmune or inflammatory conditions. These findings have renewed the enthusiasm for novel pharmacological strategies targeting numerous inflammatory conditions, which currently elude successful transfer “from bench to bedside,” such as acute lung injury, sepsis, burn, ischemia-reperfusion injury, traumatic brain injury, and kidney disease.

The abundant involvement of the complement system in human health and disease is reflected by the wide range of topics covered in this special issue of *Clinical and Developmental Immunology*.

B. Nilsson and N. Ekdahl provide a summarizing article on current indications, techniques, sampling, and interpretations for clinical complement analyses. In addition, an easy-to-follow algorithm is provided for routine laboratory operations.

G. Cazander et al. illustrate the success of targeted complement inhibition of complement in wound healing and demonstrate how revesal of complement hyperactivity using several complement inhibitors has resulted in novel and innovative wound care strategies, which may soon be subject of clinical trials. 

K. J. Welsh et al. challenge recent evidence that complement plays a critical role in the host defense against *Mycobacterium tuberculosis*. Following aerosol challenge with *Mycobacterium tuberculosis*, the authors demonstrate that “C7-knockout” mice had markedly reduced liver colony forming units and lung occlusion in conjunction with significantly increased total lymphocytes, decreased macrophages, and increased numbers of CD4+ cells. In line, expression of lung IFN-*γ* and TNF-*α* was increased in these animals, underscoring a crucial role of C7 in the disease manifestation of *Mycobacterium tuberculosis*.

S. R. Stowell et al.'s manuscript reviews the role of complement in transfusion-related mortality. This is of particular interest, as excessive complement activation during transfusion represents one of the most common features associated with fatality. The treating physician is furthermore provided with several strategies aiming to immunomodulate the complement system during incompatible red blood cell transfusions.

In two reviews, F. Bu et al. and S. F. Heeringa and colleagues review complement involvement in renal disease. Bu reviews the pathogenetic role of genetic variations in complement genes and complement dysregulation at the cell surface results in different forms of atypical hemolytic uremic syndrome, and how this understanding may result in novel complement-modulating treatment approaches. In S. F. Heeringa's paper, the pathogenesis of complement associated glomerulopathies is reviewed. Heeringa describes how defective complement control via the alternative pathway results in glomerular C3 deposition and how this understanding has generated a complement pan-blockade or plasma substitution as potential treatment strategies, which have been proven successful.

H. Nishiura et al. shed further light on the importance of the C5a receptor (C5aR) during erythropoiesis. Strikingly, expression and regulation of RP S19 oligomer, which binds to C5aR, may regulate C5aR function as a connector for the erythroblast-macrophage island or for erythroblast differentiation in the bone marrow.

D. Rittirsch et al. and J. Charchaflieh et al. review the highly elaborate immune response following trauma and sepsis, which is—at least in part—driven by excessive complement activation and may result in a deadly downward spiral of SIRS, sepsis, septic shock, and multiorgan failure. Recent clinical concepts, such as “damage control surgery,” transition such findings into the clinical setting in an attempt to improve outcome of a highly vulnerable patient population.

These manuscripts represent an exciting and insightful snapshot of current complement research. State-of-the-art, existing challenges, and emerging future topics are highlighted in this special issue, which may inspire the reader and help advance the present complement research. We would like to thank all authors, reviewers, and the Editor-in-Chief for making this special issue in *Clinical and Developmental Immunology *possible.

*Michael A. Flierl* *Daniel Rittirsch* *Markus S. Huber-Lang* *Philip F. Stahel*

